# Stable White Matter Structure in the First Three Years After Psychosis Onset

**DOI:** 10.1016/j.bpsgos.2025.100472

**Published:** 2025-02-20

**Authors:** Peter C. Van Dyken, Kun Yang, Andreia V. Faria, Akira Sawa, Michael MacKinley, Ali R. Khan, Lena Palaniyappan

**Affiliations:** a Neuroscience Graduate Program, Schulich School of Medicine & Dentistry, Western University, London, Ontario, Canada; b Department of Psychiatry, Johns Hopkins University School of Medicine, Baltimore, Maryland; c Department of Radiology, Johns Hopkins University School of Medicine, Baltimore, Maryland; d Departments of Psychiatry, Neuroscience, Biomedical Engineering, Pharmacology, and Genetic Medicine, Johns Hopkins University School of Medicine, Baltimore, Maryland; e Department of Mental Health, Johns Hopkins University Bloomberg School of Public Health, Baltimore, Maryland; f Lawson Health Research Institute, London Health Sciences Centre, London, Ontario, Canada; g Robarts Research Institute, Western University, London, Ontario, Canada; h Department of Medical Biophysics, Schulich School of Medicine & Dentistry, Western University, London, Ontario, Canada; i Douglas Mental Health University Institute, McGill University, Montreal, Quebec, Canada; j Department of Psychiatry, Schulich School of Medicine & Dentistry, Western University, London, Ontario, Canada

**Keywords:** Diffusion tensor imaging, First-episode psychosis, Longitudinal, Negative symptoms, Schizophrenia, Tractography

## Abstract

**Background:**

White matter alterations observed using diffusion weighted imaging have become a hallmark of chronic schizophrenia, but it is unclear when these changes arise over the course of the disease. Nearly all studies reported to date have been cross-sectional, so despite their large sample sizes, they cannot determine whether changes accumulate as a degenerative process or patients with preexisting white matter damage are predisposed to more chronic forms of schizophrenia.

**Methods:**

We examined 160 scans comprising 2 years of annual follow-up data from 42 control participants and 28 patients with schizophrenia recruited in the first 2 years since their diagnosis, totaling 2 to 3 scans per participant. We also examined 6-month follow-up data obtained from an ultra-high field (7T) scanner (68 scans; *n* = 19 patients with first-episode schizophrenia, *n* = 15 control participants) as a validation dataset. A longitudinal model was used to compare the trajectory of diffusion tensor parameters in patients and control participants.

**Results:**

Positive and negative symptom scores were correlated with diffusion parameters using region of interest-based approaches. No longitudinal differences between patients and control participants were observed for any diffusion tensor imaging parameter in either dataset. However, we did observe consistent associations between white matter alterations and negative symptoms in both datasets.

**Conclusions:**

White matter does not appear to be susceptible to schizophrenia-linked degeneration in the early stages of disease, but preexisting pathology may be linked to disease severity.

## Plain Language Summary

This study explored changes in brain white matter over time in early schizophrenia using an MRI modality called diffusion weighted imaging. We analyzed scans from recently diagnosed patients and healthy control participants across 2 datasets, examining whether white matter in patients degenerates over time. We did not observe white matter degeneration in early schizophrenia, but we did find connections between white matter changes and the severity of negative symptoms, such as apathy and social withdrawal, in both datasets. These findings suggest that white matter pathology may not develop over time but instead reflect preexisting abnormalities that could influence the severity of the disease.

Diffusion tensor imaging (DTI) studies of patients with chronic schizophrenia have robustly demonstrated a reduction of fractional anisotropy (FA) across the white matter, potentially reflecting increased axonal disorganization or reduced fiber integrity (1, 2, 3). However, these findings are much more limited in patients with early psychosis. In our own recent investigation, we did not find any substantial differences between patients and control participants in 2 separate datasets (4). Other studies have found differences with only limited effect size (5, 6, 7). The neurodegenerative hypothesis of white matter in schizophrenia argues that patients experience progressive deterioration of white matter over the disease course (8). It is possible that despite the lack of substantial white matter integrity at the outset, DTI metrics may begin to change after the onset of psychosis.

Previous reports have argued in favor of this white matter neurodegeneration hypothesis, citing data showing increasingly disrupted DTI metrics in people with established schizophrenia (9). However, nearly all these studies have been cross-sectional, and although they reported declining FA in chronic patients (10, 11, 12), they could not disambiguate individual changes over time from cohort effects. In other words, rather than sustaining longitudinal degeneration, patients with preexisting lower FA may selectively progress to more chronic forms of schizophrenia. Cross-sectional data are inadequate to test this alternative explanation.

Unfortunately, high-quality longitudinal studies with diffusion imaging, including both patients and control participants who were followed for multiple years, are rare. In patients with established illness (>13 years mean duration), Tronchin *et al.* (13) reported higher FA decline in patients after 6 months around the genu of the corpus callosum. A 3-year follow-up study of 55 patients (<5 years mean illness duration) by Domen *et al.* (14) found net FA decline relative to control participants in a few regions of interest (ROIs) but no global changes. In patients with first-episode psychosis (FEP), Berge *et al.* (15) failed to find an effect of time in 20 patients after a 2-year follow-up; Serpa *et al.* (16) reached a similar conclusion in a 6-month follow-up of 21 patients. Results from ultra-high-risk patients (without a formal schizophrenia diagnosis) have been heterogeneous, with 3 studies finding significant decreases in FA compared with control participants (17, 18, 19), one study finding a significant increase over 6 months (20), and 3 studies not finding any differences (21, 22, 23).

Studies of a few datasets with follow-up times <6 months have been published. Two of these, one with patients with chronic psychosis (24) and one with patients with early psychosis (25,26), did not find any longitudinal differences. One 6-week study of patients with FEP showed increasing FA in 1 ROI relative to healthy control participants (HCs) (27). Studies of another 6-week dataset of patients with FEP found widespread FA decline (28,29). All other studies reviewed were limited by lack of HC follow-up (30, 31, 32, 33, 34, 35) or a lack of angular resolution in the diffusion sequence (≤10 directions) (32,33,36,37) that precluded a robust solution for the diffusion tensor that was sufficiently sensitive to within-individual changes (38,39). In sum, short-term longitudinal studies seem to produce inconsistent observations, irrespective of the stage of illness. While a minimum necessary follow-up period to observe longitudinal changes is difficult to establish, in first-episode cohorts, both symptomatic and functional stabilization appear to occur over a 6-month treatment period (40, 41, 42).

In HCs, FA peaks in most regions by around age 30 before gradually declining with age (43, 44, 45, 46). Previous authors have suggested that this age-related decline starts prematurely in patients with schizophrenia during the latter phases of white matter maturation, resulting in lower FA across adult life despite an absolute rate of age-related FA decline similar to that in HCs (8,9). Thus, this early period is likely the most important for detecting individual effects of FA trajectory. With only 5 relatively small published datasets having investigated such patients to date, we cannot definitively say whether this early peak exists. While youths at high risk for psychosis have been followed in larger studies (18), these patients typically do not convert to schizophrenia (<15%–20% over several years), and studies have not had sufficient follow-up times to begin to detect degenerative decline.

In this report, we address this gap with findings from the JHSZC (Johns Hopkins Schizophrenia Center) dataset, a 3-year follow-up study of patients with early psychosis and HCs with 3 time points for most participants. We also validate our analysis with 6-month follow-up data from the TOPSY (Tracking Outcomes in Psychosis) dataset that has been reported on previously (4). Because the age of our participants is close to the early-peak period of developmental risk (9), we expected to find the characteristic pattern of white matter changes reported in the largest cross-sectional study of psychosis to date (1): progressive FA decline with increasing mean diffusivity (MD), radial diffusivity (RD), and axial diffusivity (AD) compared with HCs. If confirmed, this may indicate early progressive changes in both myelin and axonal integrity in schizophrenia.

## Methods and Materials

### Data

#### Johns Hopkins Schizophrenia Center

This study was approved by the Johns Hopkins Medicine Institutional Review Board. A total of 101 patients and 96 HCs were recruited from within Johns Hopkins Hospital and the surrounding region. All study participants provided written informed consent. Patients were treated with antipsychotic therapy and specialized case management as needed. All participants were between 13 and 35 years of age and had no significant neurological history, no previous drug or alcohol abuse, and no illicit drug use within 2 months of study onset. Patients were within 24 months of psychosis onset. All but 6 patients were already medicated at their first (baseline) visit. The 6 medication-naïve patients started medication during the follow-up period. The study psychiatrists did not make treatment decisions regarding medications. Participants diagnosed with bipolar disorder with psychotic features (*n* = 22), major depressive disorder with psychotic features (*n* = 4), substance-induced psychotic disorder (*n* = 3), or psychosis not otherwise specified (*n* = 3) were excluded.

Follow-up assessments for both HCs and patients were obtained where possible at 1, 2, and 3 years from baseline.

Data were acquired with a head-only, neuro-optimized 3T magnetic resonance imaging (MRI) scanner (Philips Intera version 3.2.2). T1-weighted (T1w) data were collected using an magnetization-prepared rapid acquisition gradient-echo (MPRAGE) sequence at 1-mm isotropic resolution, echo time (TE) = 3.7 ms, repetition time (TR) = 8.1 seconds, field of view (FOV) = 224 × 180 × 165 mm, number of slices = 165, flip angle = 8. Two replicate diffusion datasets were acquired per participant per session with an echo planar imaging (EPI) sequence at 0.74 × 0.74 × 2.2 mm resolution, TE = 75 ms, TR = 7.4 seconds, FOV = 212.53 mm, number of slices = 70, multiband (MB) acceleration factor = 2, flip angle = 90. Thirty-two directions were acquired in the anteroposterior (AP) direction at b = 700 together with 1 b = 0 images.

Baseline, but not follow-up, diffusion data from this dataset have been reported previously (7,47,48).

#### Tracking Outcomes in Psychosis

A total of 71 patients with FEP and 39 HCs were recruited from an established cohort of individuals enrolled in PEPP (Prevention and Early Intervention Program for Psychoses), a high-fidelity early intervention program that serves all patients with FEP in the catchment area of London, Ontario. Patients were treated with antipsychotic therapy and specialized case management as needed. All participants provided written, informed consent prior to participation. Patients with FEP included individuals experiencing their first psychotic episode and who had no more than 14 days of cumulative lifetime antipsychotic exposure. HCs had no history of mental illness, current medication use, or first-degree relatives with psychosis; they were group matched to the FEP cohort on age and sex. Individuals with major head injuries, neurological disorders, and concurrent substance use disorder were excluded from both groups.

Follow-up assessments for both HCs and patients were obtained where possible at 6 months and at 1, 2, and 3 years postbaseline. Only 1 follow-up was attained for any of our HCs; thus, analyses were conducted using data from the baseline and the first follow-up assessment for all participants.

Data were acquired with a head-only, neuro-optimized 7T MRI (Siemens MAGNETOM Plus). T1w data were collected using an MP2RAGE sequence (49) at 0.75-mm isotropic resolution, TE = 2.83 ms, TR = 6 seconds, FOV = 240 × 240 mm, number of slices = 208. The T1w image was reconstructed using the robust algorithm introduced by O’Brien *et al.* (50). Diffusion data were acquired with an EPI sequence at 2-mm isotropic resolution, TE = 50.2 ms, TR = 5.1 seconds, FOV = 208 mm, number of slices = 72, MB acceleration factor = 2, flip angle = 90. Sixty-four directions were acquired in both the AP and posteroanterior (PA) directions at b = 1000, along with 2 b = 0 images. Gradient nonlinearity correction was applied to all acquisitions using in-house software.

Baseline, but not follow-up, diffusion data from this dataset has been previously reported (4).

Complete inclusion/exclusion criteria are described in Supplemental Methods.

### Preprocessing

#### Diffusion Data

As in (4), diffusion data were preprocessed using *snakedwi* (51), a preprocessing pipeline based on *snakebids* (52) and *snakemake* (53). Briefly, Gibbs ringing artifacts were removed with *mrdegibbs* from *MRtrix3* (54,55); eddy currents and motion were corrected using *eddy* from FSL (56); susceptibility-induced distortions were corrected using *topup* in FSL, using the AP/PA pairs of images (57,58). The T1w image was skull stripped with *SynthStrip* (59); bias field correction was applied with *N4ITK* from ANTS (60). A T1w proxy image was created from the diffusion image using *SynthSR* (61) and used to accurately register the diffusion image space to the T1w space using a rigid transform calculated with *greedy* (62). DTI metrics were calculated using *dtifit* from FSL with linear regression (63).

#### JHSZC Preprocessing

JHSZC data were preprocessed as with TOPSY, with the following changes:

The replicate diffusion acquisitions were concatenated before processing.

For susceptibility distortion correction without a reverse phase–encoding scan, the T1w image was first rigid transformed to the diffusion b0 image. A T1w proxy image was created from the diffusion b0 images using *SynthSR* (61) and registered to the aligned T1w image with *antsRegistration* (64). The inverse warp field from this registration was used to unwarp the distorted diffusion images.

Slice to volume correction in *eddy* was used to correct intravolume motion correction. Following correction, all diffusion volumes were visually inspected for uncorrected motion artifacts (e.g., zebra-striping). Volumes with qualitatively severe artifacts were manually excluded. Because of the replicate datasets, this exclusion did not cause bias in the sampling directions.

### Parcellations

FA maps were first nonlinearly registered to a common template corresponding to the average space of all FA images (62,65) (see Supplemental Methods). An FA skeleton was derived using FSL (66). FA, MD, AD, and RD values were projected to this skeleton, then sampled with a composite atlas comprising the Johns Hopkins University (JHU) white matter atlas, capturing core white matter regions, and the Talairach lobe segmentation, capturing peripheral white matter regions. To increase our sensitivity to local and global changes, the ROIs were organized into a hierarchical system as previously described (67) (see Supplemental Methods for a rigorous description), with the following levels: 1) a single ROI covering the entire white matter skeleton; 2) core and peripheral regions; 3) peripheral lobe ROIs derived from the Talairach segmentation and 4 groupings of the JHU atlas labeled as projection, association, callosal, and limbic tracts (see Table S2); 4) individual JHU ROIs. Multiple comparisons were corrected separately for each level using the Benjamini-Hochberg false discovery rate (FDR) procedure (68).

### Analysis

Longitudinal analysis was performed with a linear mixed-effects model [R version 4.3.3 (69)] using *lme4* (version 1.1.35.3) (70). Random intercepts and slopes were fit for each participant (only random slopes for the TOPSY dataset, which had just 2 time points); age and sex were regressed:

(1) P∼session+group+session:group+age+sex+(session|subject)

The significance of the random slopes and intercepts was tested using *ranova* from *lmerTest* (version 3.1.3) (71). Degrees of freedom for each comparison were estimated using Satterthwaite’s method (72) with *lmertest* (version 3.1.3) (71). One-way comparisons were used to test for greater FA reductions, with MD, AD, and RD increases in patients versus control participants.

For clinical score analyses, positive and negative symptoms were measured in the JHSZC using the Scale for the Assessment of Positive Symptoms (SAPS) and Scale for the Assessment of Negative Symptoms (SANS) (73) and in TOPSY using positive and negative subscores from the 8-item Positive and Negative Syndrome Scale (PANSS-8) (74,75). We included all patients with valid clinical score measurements from at least 2 sessions and at least 1 valid scan. For each score and participant, the intercept and slope of the score across time were computed using a first-order polynomial. For TOPSY, we also split the participants into 2 groups according to whether these clinical scores were at a minimum value (i.e., *N1* + *N4* + *N6* = 3; *P1* + *P2* + *P3* = 3) at the second session. The minimal-score group was called the remission group for the positive or negative symptom domain. Sampled ROIs were averaged across sessions for each participant. ROI statistics were generated using *t* tests controlling for age and sex. One-way comparisons were used to test the hypothesis that lower FA and higher MD, AD, and RD correlated with higher score slope and intercept. For remission, we tested the hypothesis that FA would be higher and MD, AD, and RD would be lower in the remission group.

## Results

### Demographics

In the JHSZC dataset (Table 1), 50 HCs and 40 patients were lost to follow-up (Table S5). Four additional HCs and 1 patient did not have usable diffusion data. This left 42 HCs and 28 patients from the JHSZC dataset. Two session-specific scans were dropped due to quality, but the affected participants still had 2 usable sessions included in the study (4 subjects had no baseline scan due to a failed protocol or poor quality, but did have 2 follow-ups). The number of dropouts per group did not vary (χ^2^_1_ = 1.17, *p* = .28), and the distribution of HCs and patients across included sessions did not vary (χ^2^_2_ = 0.41, *p* = .81).

**Table 1:** JHSZC Demographics

	Healthy Control Group, *n* = 42			Early Psychosis Group, *n* = 28^a^			
	Baseline, *n* = 42	1 Year, *n* = 37	2 Years, *n* = 18	Baseline, *n* = 24	1 Year, *n* = 25	2 Years, *n* = 11	3 Years, *n* = 1
Sex, Female/Male	20/22	17/20	9/9	5/19	5/20	1/10	0/1
Age, Years	23.93 (3.39)	25.30 (3.39)	25.72 (3.48)	22.08 (3.75)^b^	23.04 (3.82)^b^	22.09 (3.11)^b^	24.00
Ethnicity							
Black/African	23	20	10	17	19	8	1
East Asian	2	1	1	0	0	0	0
Other/unknown	2	2	1	1	1	0	0
White/European	15	14	6	6	5	3	0
Handedness, Right/Left	35/7	30/7	17/1	21/3	22/3	9/2	1/0
Smoker, Yes/No	3/39	4/33	1/17	5/19	12/13^b^	5/6^b^	0/1
Cannabis, Yes/No	3/39	4/32	2/16	4/20	6/19	0/11	0/1
Duration of Illness, Weeks^c^	–	–	–	65.00 (55.25)	121.33 (47.67)	160.33 (21.67)	255.67
CPZ, mg	–	–	–	234.58 (209.51)	270.76 (283.29)	336.36 (285.56)	250.00
SAPS	–	–	–	4.35 (4.35)	4.52 (2.27)	3.73 (4.78)	0.00
SANS	–	–	–	7.96 (3.75)	7.52 (3.59)	7.91 (6.02)	0.00

Values are presented *n* or mean (SD) unless otherwise specified.

CPZ, chlorpromazine equivalent dose; JHSZC, Johns Hopkins Schizophrenia Center; SANS, Scale for the Assessment of Negative Symptoms; SAPS, Scale for the Assessment of Positive Symptoms.

a Four subjects had no baseline scan due to a failed protocol or poor quality.

b Values in the early psychosis group are significantly different relative to the same session in healthy control participants.

c Median (IQR).

There was a significantly greater proportion of males than females at each session in the patient group than the control group, and control participants were significantly older than patients across the study. Patients who returned for their 1-year follow-up were significantly more likely to smoke than their healthy counterparts (Table S3). Following the baseline scan, initial follow-ups were at 1.1 ± 0.1 years. Second follow-ups were at 2.1 ± 0.2 years. Follow-up times did not differ between patients and HCs for the first (*t*_62_ = 0.29, *p* = .77) or second (*t*_30_ = 0.93, *p* = .36) follow-up (Figure S1A).

In the TOPSY dataset (Table 2), 23 HCs and 49 patients were lost to follow-up (Table S6). One HC and 3 patients did not have usable diffusion data from each session, which left 15 HCs and 19 patients for analysis. The number of dropouts did not vary significantly between groups (χ^2^_1_ = 0.34, *p* = .56). Second follow-ups were obtained for 4 patients but not for any control participants; accordingly, these data were excluded from the study.

**Table 2:** TOPSY Demographics

	Healthy Control Group, *n* = 15		First-Episode Psychosis Group, *n* = 19	
	Baseline, *n* = 15	6 Months, *n* = 15	Baseline, *n* = 19	6 Months, *n* = 19
Sex, Female/Male	5/10	5/10	3/16	3/16
Age, Years	21.73 (2.99)	22.60 (3.04)	22.53 (5.42)	23.11 (5.40)
Ethnicity				
Black/African	0	0	2	2
Caribbean/North American Black	0	0	1	1
East Asian	5	5	0	0
White/European	10	10	16	16
Handedness, Right/Left/Ambidextrous	14/0/1	14/0/1	18/0/1	18/0/1
Education, Years	14.27 (2.02)	14.33 (2.09)	12.95 (1.18)^a^	12.95 (1.18)^a^
Socioeconomic Status	3.20 (1.57)	3.20 (1.57)	3.89 (1.08)	3.89 (1.08)
CAST	7.00 (3.87)	13.50 (10.61)	12.12 (6.90)^a^	NA
AUDIT-C	2.87 (2.26)	5.00 (2.83)	3.79 (3.83)	NA
Smoker, Yes/No	1/14	1/14	7/12	0/19
Cannabis, Yes/No	5/10	5/10	12/7	12/7
SOFAS	81.08 (6.24)	79.71 (5.25)	42.00 (13.37)^a^	61.89 (14.68)^a^
Duration of Illness, Weeks	–	–	104.00 (132.50)^b^	130.07 (140.18)^b^
Antipsychotic Day of Scan, DDD	–	–	0.00 (0.33)^b^	1.00 (0.66)^b^
Antipsychotic Lifetime, DDD × Days	–	–	0.00 (1.25)^b^	135.64 (107.44)^b^
CDS	–	–	0.58 (0.51)	0.21 (0.42)
PANSS-8				
Total	–	–	24.68 (5.53)	13.53 (4.57)
Positive	–	–	12.53 (2.82)	4.95 (1.93)
Negative	–	–	6.26 (3.31)	5.95 (3.58)
General	–	–	5.89 (2.54)	2.53 (1.12)

Values are presented mean (SD) or *n* unless otherwise specified.

AUDIT-C, Alcohol Use Disorders Identification Test–Consumption; CAST, Cannabis Abuse Screening Test; CDS, Calgary Depression Scale; DDD, defined daily dose; NA, not applicable; PANSS-8, 8-item Positive and Negative Syndrome Scale; SOFAS, Social and Occupational Functioning Assessment Scale; TOPSY, Tracking Outcomes in Psychosis.

a Values in the first-episode psychosis group are significantly different relative to the same session in healthy control participants.

b Median (IQR).

HCs and patients were group matched on age, sex, and socioeconomic status. Patients had significantly lower levels of education, a higher Cannabis Abuse Screening Test score [indicating increased risk of cannabis abuse (76)], and a lower Social and Occupational Functioning Assessment Scale score (77) (Table S4). There was no correlation between the Calgary Depression Score and the PANSS8-Negative (PANSS-8N) subscore. Follow-ups took place 0.6 ± 0.3 years following the baseline scan. HCs had significantly longer follow-up times than patients (*t*_35_ = 2.44, *p* = .020) (Figure S1B).

### DTI Metrics Remain Stable Across Time

**Figure 1:**
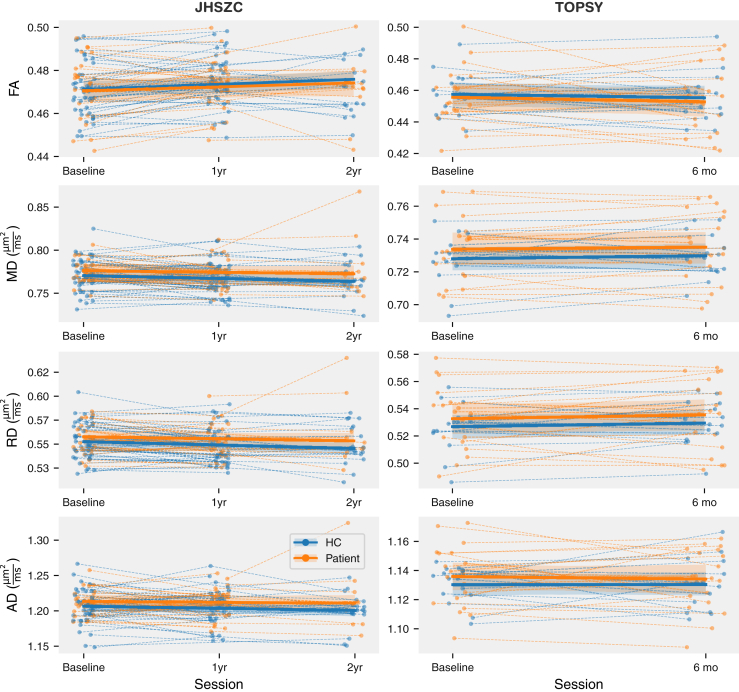
Global longitudinal changes of white matter microstructure in patients with early schizophrenia. Trendlines show a linear mixed-effect model of parameter against session with random intercepts fit for every participant. Shaded bands show a 95% CI computed with parametric bootstrapping resampling residuals and random effects 1000 times. No significant differences were found between the slopes of healthy control participants (HCs) and patients in either dataset for any of the parameters measured. In the Johns Hopkins Schizophrenia Center (JHSZC) sample, fitting random slopes to each participant did not significantly improve the fit of the model (not tested in Tracking Outcomes in Psychosis [TOPSY] because each participant had only 2 time points). AD, axial diffusivity; FA, fractional anisotropy; MD, mean diffusivity; RD, radial diffusivity.

A mixed linear-effects paradigm was used to model longitudinal DTI parameter changes. Because only 1 participant in the JHSZC dataset had a third follow-up scan, this session was not used in this longitudinal analysis. No significant differences in slope between HCs and patients were observed for FA, MD, AD, or RD in any ROI after correcting for multiple comparisons (Figure 1).

A significant increase in global FA (2-tailed test; *t*_131.26_ = 2.043, *p* = .043) and a decrease in RD (*t*_128.509_ = −1.977, *p* = .050) was observed across all participants in the JHU dataset, with localized FA increases and decreases of MD, RD, and AD in core and peripheral ROIs. Localized decreases in FA were found in the anterior internal capsule and genu of the corpus callosum (Figure S2).

Because age and sex distribution differed between HCs and patients in the JHSZC dataset, we performed a subanalysis on participants who were younger than 24 years. The resulting groups did not differ significantly on age or sex (Tables S7 and S8). No differences in longitudinal change were found between groups. The global increase and decrease of FA and RD were also no longer observed (Figure S3). We also performed a subanalysis in which we also controlled for cannabis use. The results were unchanged from the main analysis, except that the global population-wide increase in FA was no longer observed (although localized longitudinal changes in all DTI parameters remained).

### DTI Metrics in JHSZC

**Figure 2:**
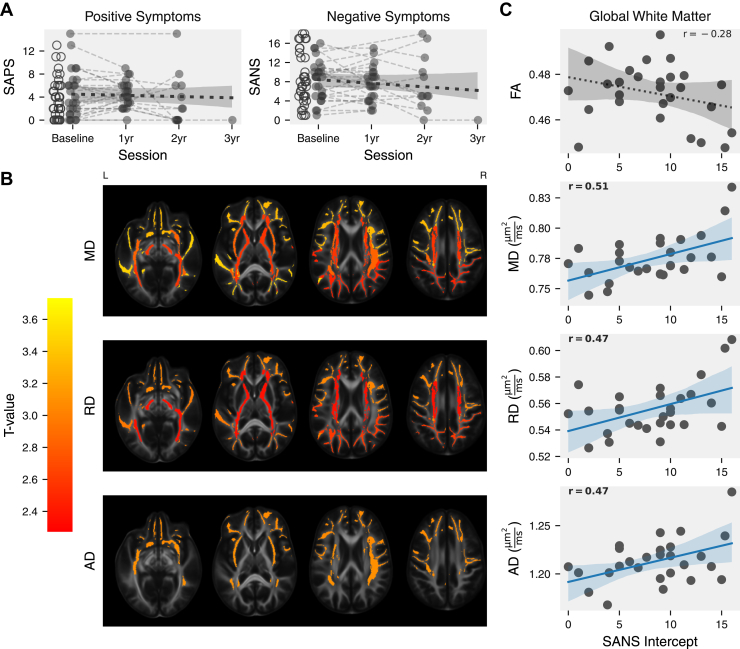
Correlation between diffusion tensor imaging (DTI) parameters and negative symptom (Scale for the Assessment of Negative Symptoms [SANS]) intercepts in the Johns Hopkins Schizophrenia Center dataset. DTI parameters were averaged across all sessions for each participant. **(A)** Longitudinal progression of symptoms. Empty circles at baseline show participants with no follow-ups. Trendlines show a linear fixed-effect model of parameter against session with random slopes and intercepts fit for every participant. Shaded bands show a 95% CI computed with parametric bootstrapping resampling residuals and random effects 1000 times. Neither parameter significantly varied with session (Scale for the Assessment of Positive Symptoms [SAPS] *t*_22.9_ = −0.43, *p* = .66) (SANS *t*_27.3_ = −0.92, *p* = .36). **(B, C)** Intercept was computed using a first-order linear model for each participant, with the baseline scan as time 0. Relationships with DTI parameters were tested with a linear model with age and sex and covariates. **(B)** Significant regions of interest are colored according to their *t* value. Multiple comparisons were corrected with the false discovery rate. **(C)** Scatter plots showing DTI parameters averaged across the white matter. Shaded bands show 95% CI computed with nonparametric bootstrap paired resampling with 1000 permutations. Mean diffusivity (MD), radial diffusivity (RD), and axial diffusivity (AD) significantly increased with session. Fractional anisotropy (FA) did not significantly change. *t* Values and *p* values are shown in Table S10.

Next, we investigated whether SAPS and SANS corresponded with DTI metrics. In the JHSZC dataset, comprising patients who enrolled after the start of treatment, neither clinical score showed significant change across sessions (Figure 2A). The SANS intercept was significantly correlated with the global average of DTI metrics: Higher symptom severity corresponded with higher MD, RD, and AD (Figure 2C). These associations were reproduced in the core and peripheral white matter ROIs. Associations of MD, RD, and AD with the SANS intercept were observed in several more discrete ROIs, varyingly including the frontal, parietal, and temporal lobes, and the association and projection ROI groups, as well as several JHU atlas ROIs (see Figure 2B, Figure S5, and Table S10). FA had no significant associations. No parameter in any ROI had an association with the SANS slope or the SAPS slope or intercept. These results were replicated using the Tract-Based Spatial Statistics (TBSS) model (Figure S6).

### DTI Metrics in TOPSY

**Figure 3:**
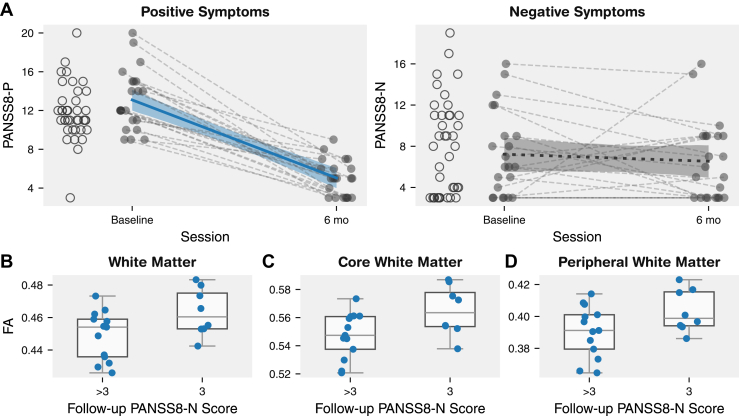
Correlations between diffusion tensor imaging (DTI) parameters and the 8-item Positive and Negative Syndrome Scale-Negative (PANSS8-N) follow-up score in the Tracking Outcomes in Psychosis dataset. DTI measures were averaged across sessions per participant. **(A)** Longitudinal progression of symptoms. Empty circles at baseline show participants with no follow-ups. Trendlines show a linear fixed-effect model of parameter against session with random intercepts fit for every participant. Shaded bands show a 95% CI computed with parametric bootstrapping resampling residuals and random effects 1000 times. PANSS8 Positive (PANSS8-P) was significantly lower at the second session (*t*_21.0_ = −10.9, *p* < .001). PANSS8-N did not significantly change (*t*_21.0_ = −0.70, *p* = .49). **(B–D)** Participants grouped based on whether their PANSS8-N score at follow-up was equal to 3, the lowest possible score (remission). Relationships with DTI parameters tested with a linear model with age and sex and covariates. Regions of interest from each panel come from different nested hierarchical layers at successively higher resolutions. Multiple comparisons for each layer were corrected with the false discovery rate. All comparisons shown are significant. *t* Values and *p* values are shown in Table S9. FA, fractional anisotropy.

We repeated the above analyses in the TOPSY dataset using PANSS8-Positive (PANSS8-P) and PANSS8-N. We included 2 additional participants who had valid clinical scores but were missing scans at their second session, for a total *n* = 21. Because only a few participants had clinical score data for more than 2 sessions (Figure S4), we used only the baseline and first follow-up score from each participant. (Figure 3A). The remission:nonremission split for PANSS8-P was 7:14 and for PANSS8-N was 8:13.

Significantly higher global FA was found in the PANSS8-N remission group (Figure 3B). The finding was replicated in both the core and peripheral white matter following FDR correction (Figure 3C), as well as in frontal, parietal, and temporal regions and in association fiber ROIs from the JHU atlas. No effect on FA was found for the PANSS8-N slope or intercept or for any of the PANSS8-P metrics. No effects were found for any other DTI parameter. These results were replicated using the TBSS and network-based statistic models (Figures S6 and S7).

## Discussion

In this study, we failed to find significant longitudinal effects in DTI parameters in patients with early schizophrenia from 2 different datasets obtained at different field strengths and recruited within 3 years of diagnosis. Although our results cannot disprove the neurodegenerative hypothesis for white matter in psychosis, we note that FA in patients putatively reaches an early developmental peak in their twenties (9), so if any longitudinal differences of DTI parameter slopes are to be found between patients and control participants, we would reasonably expect them to appear within the time frame studied.

We observed a sample-wide increase in FA with time across the white matter in the JHSZC dataset. This is consistent with the healthy aging and development literature, which reports a peak of FA at around age 30 (43,46). No time effects were noted in the TOPSY dataset, likely because the shorter follow-up duration was insufficient to allow observation of this developmental effect.

Our study is consistent with most previous longitudinal studies of patients with early psychosis (15,16,26), with only one study showing relative FA increases (27) and another finding decline (29). As with most of these studies, however, our work is limited by small sample sizes, and the associated lack of power remains the most likely reason for our negative results. As more data accumulate, meta-analyses may be able to pool data from these studies to obtain more reliable estimates.

Furthermore, our patients might have been too young for neurodegenerative white matter changes to occur, because disease-driven decline may not manifest until closer to the developmental peak at around 30 years of age (9). Notably, longitudinal studies of older, chronic patients have observed localized declines in FA (13,14).

The model used to describe the diffusion signal might also have contributed to study heterogeneity. A recent multicenter study found widespread reductions in FA after correcting for extracellular free water, although this study was of undiagnosed individuals at clinical high risk for schizophrenia (17). However, a longitudinal study of patients with FEP using neurite orientation dispersion and density imaging, which also accounts for free water, failed to find any longitudinal differences over a 6-week study period (25).

Finally, our study might not have been long enough to detect FA decline, which normally ranges from 0.1% to 0.5% annualized change (78). However, we note that increased follow-up time will not necessarily increase experimental power. FA in patients cannot perpetually decline faster than HCs, because this would result in implausibly low FA in old age. Thus, rates of change in the 2 groups must eventually equilibrate as FA reaches its lower plateau. It is not clear when this equilibration happens, because quadratic models fit to cross-sectional data tend to give unreliable rate estimates (79).

Although no prominent group differences were observed, we did find associations between DTI and negative symptoms in both datasets, consistent with our prior observations (4). In the JHSZC dataset, all 4 DTI parameters had spatially extensive associations with the SANS intercept. The absence of a correlation with the SANS slope suggests either that treatment response is independent from changes in white matter integrity or that the scale of symptom progression across the JHSZC dataset was insufficient to relate to white matter, especially because these patients were more established on their treatments than those in the TOPSY cohort.

Negative symptoms are often secondary to, and ameliorate with treatment of, positive symptoms (80). In our TOPSY dataset, where positive symptoms in most patients responded well to treatment, and depressive episodes were ruled out via clinical consensus, residual negative symptoms in the second session may represent persistent primary symptoms. If so, our treatment response grouping distinguished patients with and without primary negative symptoms, finding more chronic-like DTI parameters in the primary symptom group. Previous studies have in fact shown that patients with deficit schizophrenia, defined by the prominence of primary negative symptoms, have lower FA than nondeficit patients (81, 82, 83, 84) [but see in contrast (85)].

### Limitations

In the JHSZC dataset, patients were not matched to control participants on age or sex, both of which are known to affect DTI parameters. Analysis with an age- and sex-matched subset of the dataset also failed to find different longitudinal trajectories, but this might have been affected by the smaller sample size. Longitudinal designs are subject to attrition bias, and more severely ill patients may be less likely to remain in the study; however, we found no association between dropout and clinical scores. Our investigations of clinical symptoms were exploratory and thus used liberal significance thresholds. FDR was used to correct for multiple comparisons in our ROI analysis rather than the familywise error rate method, and no correcting was done across different parameter–clinical score combinations. Finally, we were unable to account for the effect of medication and cannabis dose on these associations. Nevertheless, the lack of changes in our sample cannot be fully explained by the fact that our patients were on antipsychotic treatments. Longitudinal reduction in FA has been reported previously in medicated patients with established illness (13,14). High-dose cannabis exposure seen among patients is expected to reduce white matter integrity (86), but this was not observed in our dataset irrespective of diagnosis.

### Conclusions

Although we did not find evidence for longitudinal change of white matter integrity in the first few years of psychosis, we did observe complementary associations between white matter state and negative symptoms in 2 completely independent datasets, which drew patients from different geographic regions and used different acquisition protocols and clinical scales. Thus, our data neither prove nor disprove a neurodegenerative hypothesis, but they do support a mediating effect of white matter on illness severity, especially negative symptom burden. More longitudinal data with longer follow-up times are needed to clarify these questions further.

## Supplementary Material

Supplemental Methods, Figures S1–S7, and Tables S1–S11

Statistics Tables

Key Resources Table
