# Supplementary material for: Stable White Matter Structure in the First Three Years After Psychosis Onset
Source: Biol Psychiatry Glob Open Sci. 2025 Feb 20;5(3):100472. doi: 10.1016/j.bpsgos.2025.100472 (PMC11994302; doi:10.1016/j.bpsgos.2025.100472)
Supplement: Supplemental Methods, Figures S1–S7, and Tables S1–S11 [file mmc1.pdf]

SUPPLEMENTARY INFORMATION

Stable White Matter Structure in the First Three Years After Psychosis Onset

Van Dyken *et al.*

1 Supplementary Figures

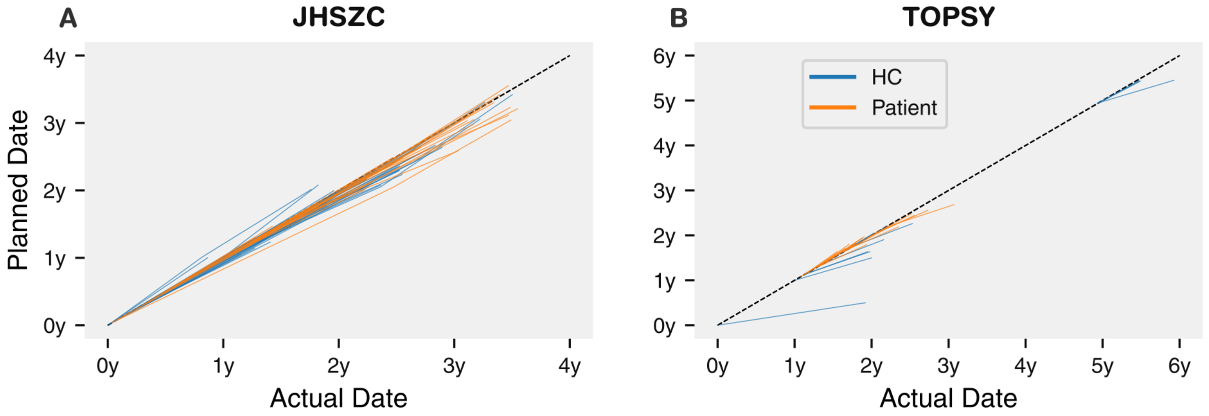

Figure S1: Actual scan dates versus target dates. 0yr corresponds to the scan date of the first subject recruited to the dataset. Time on the x- and y-axis is indicated relative to that date. The dashed, black line shows the expected interval for subjects scanned at the protocol-specified frequency: 1yr for JHSZC and 6 months for TOPSY. Coloured line segments represent the actual scan dates for individual subjects. Segments with shallower slopes than the dashed black line reflect longer than expected scan-scan intervals; steeper represent shorter intervals.

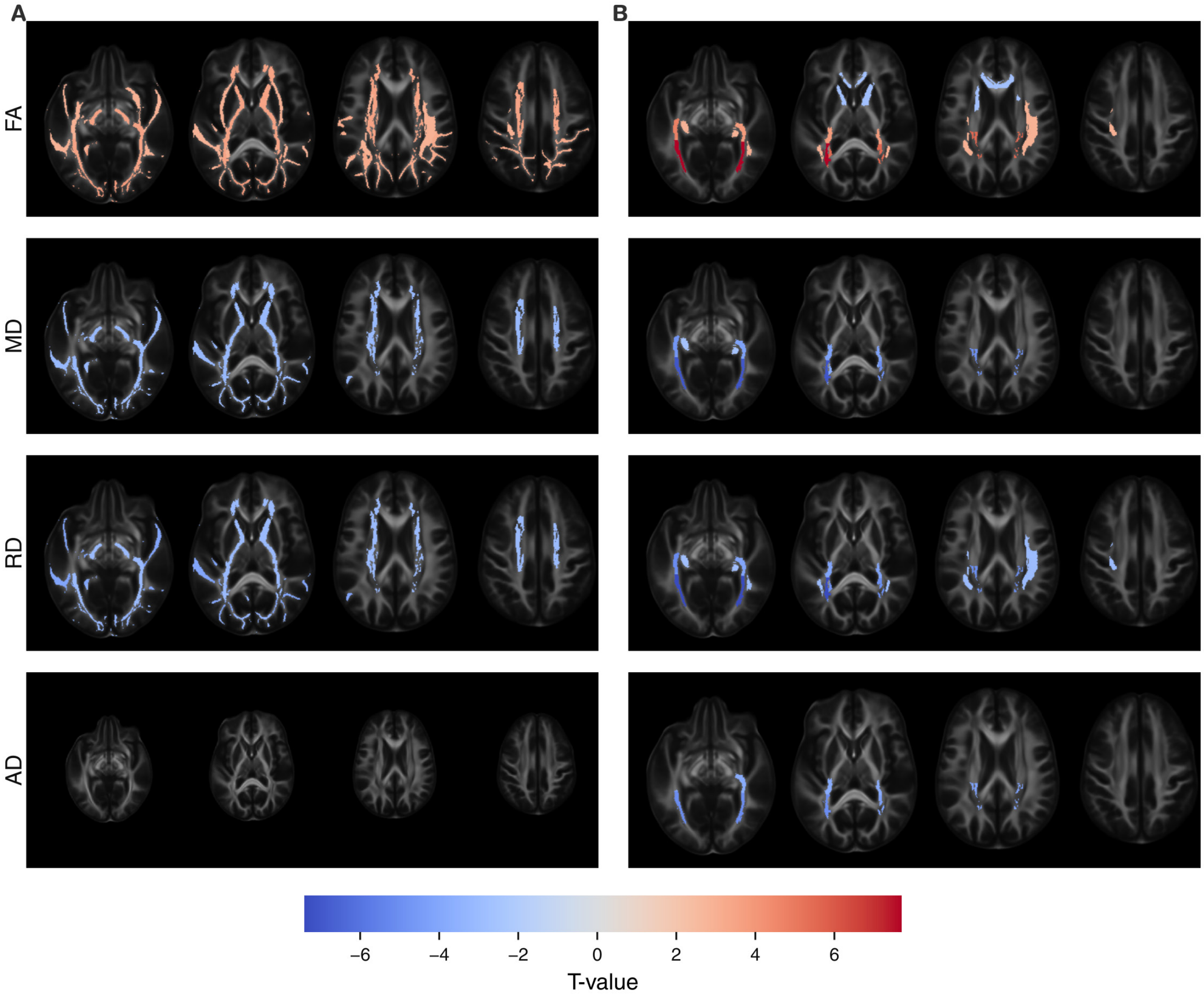

Figure S2: Main effect of time on DTI parameters in JHSZC dataset. Regions shown are significant to $p<0.05$ following FDR correction. Red-shaded regions show increases with time, blue-shaded show decreases. ***A****: Peripheral ROIs and grouped JHU atlas ROIs.* ***B****: Individual JHU atlas ROIs.*

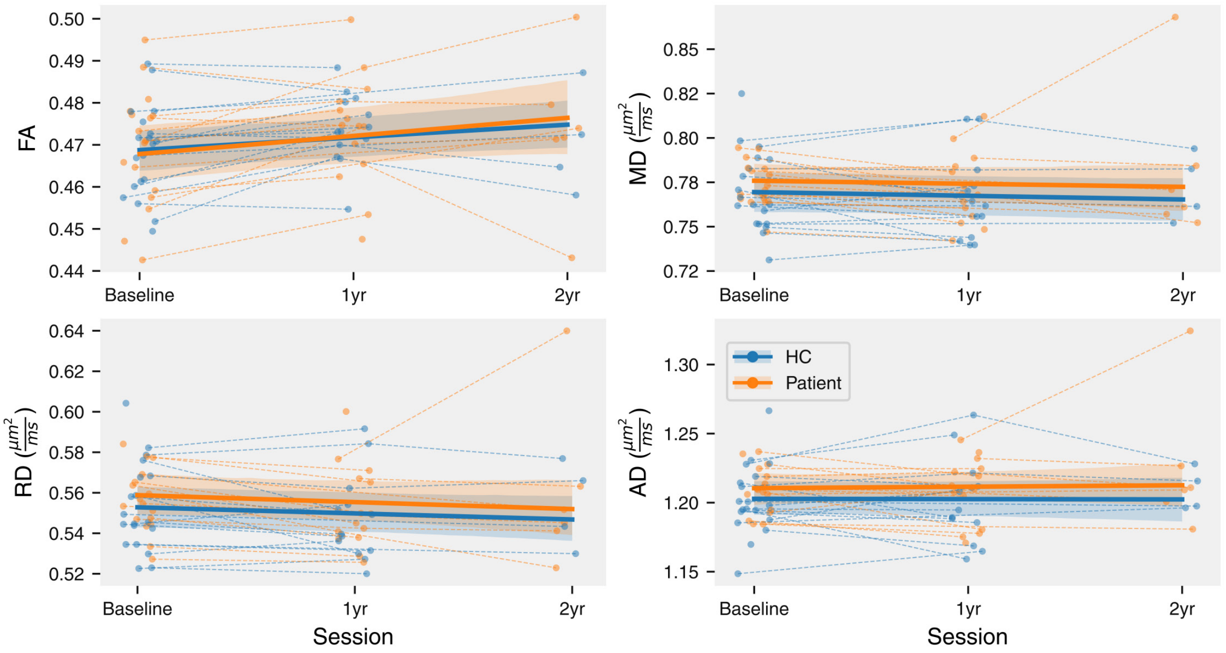

Figure S3: Global longitudinal changes of white matter microstructure in age-matched subset of JHSZC dataset. Trendlines show a linear mixed effect model of parameter against session with random intercepts fit for every subject. Shaded bands show a 95% CI computed with parametric bootstrapping resampling residuals and random effects 1000 times. No significant differences were found between the slopes of HCs and patients for any of the parameters measured. Fitting random slopes to each subject did not significantly improve the fit of the model.

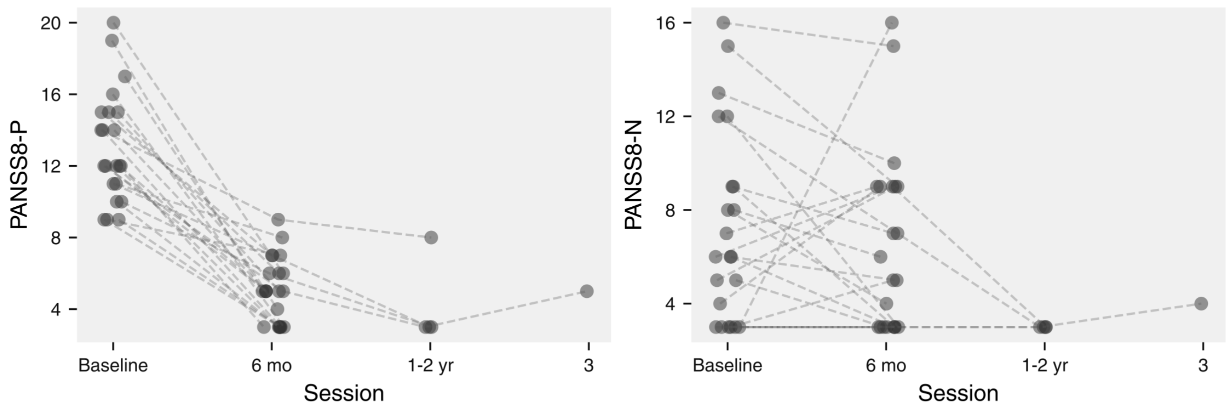

Figure S4: Clinical scores from TOPSY patients across all sessions. Each dashed line corresponds to a different subject.

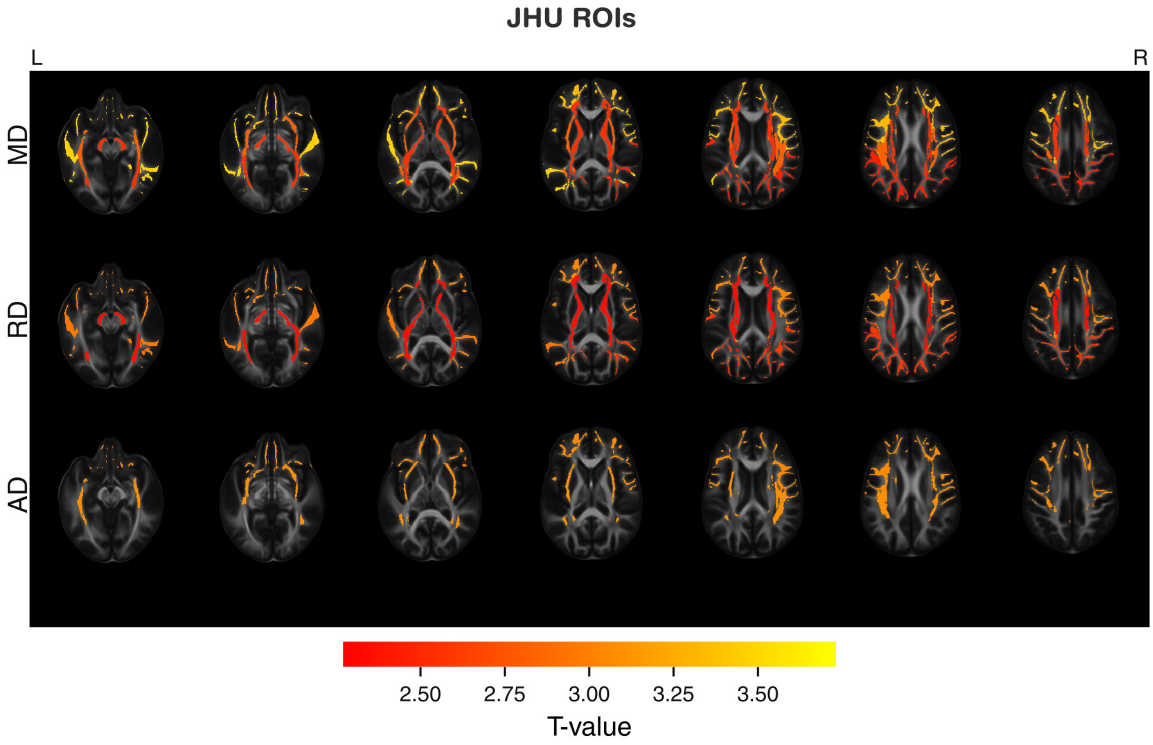

Figure S5: Correlations in the JHU atlas between microstructural parameters and SANS intercept in the JHSZC dataset. Microstructure meausures were averaged across all scans per subject, intercepts were computed with a first order linear model with the baseline session as 0. Relationships were tested with a linear model with age and sex and covariates. Significant ROIs are coloured according to their T-value. Comparisons within each layer were corrected for multiple comparisons using FDR. T-values and P-values are shown in Table S10.

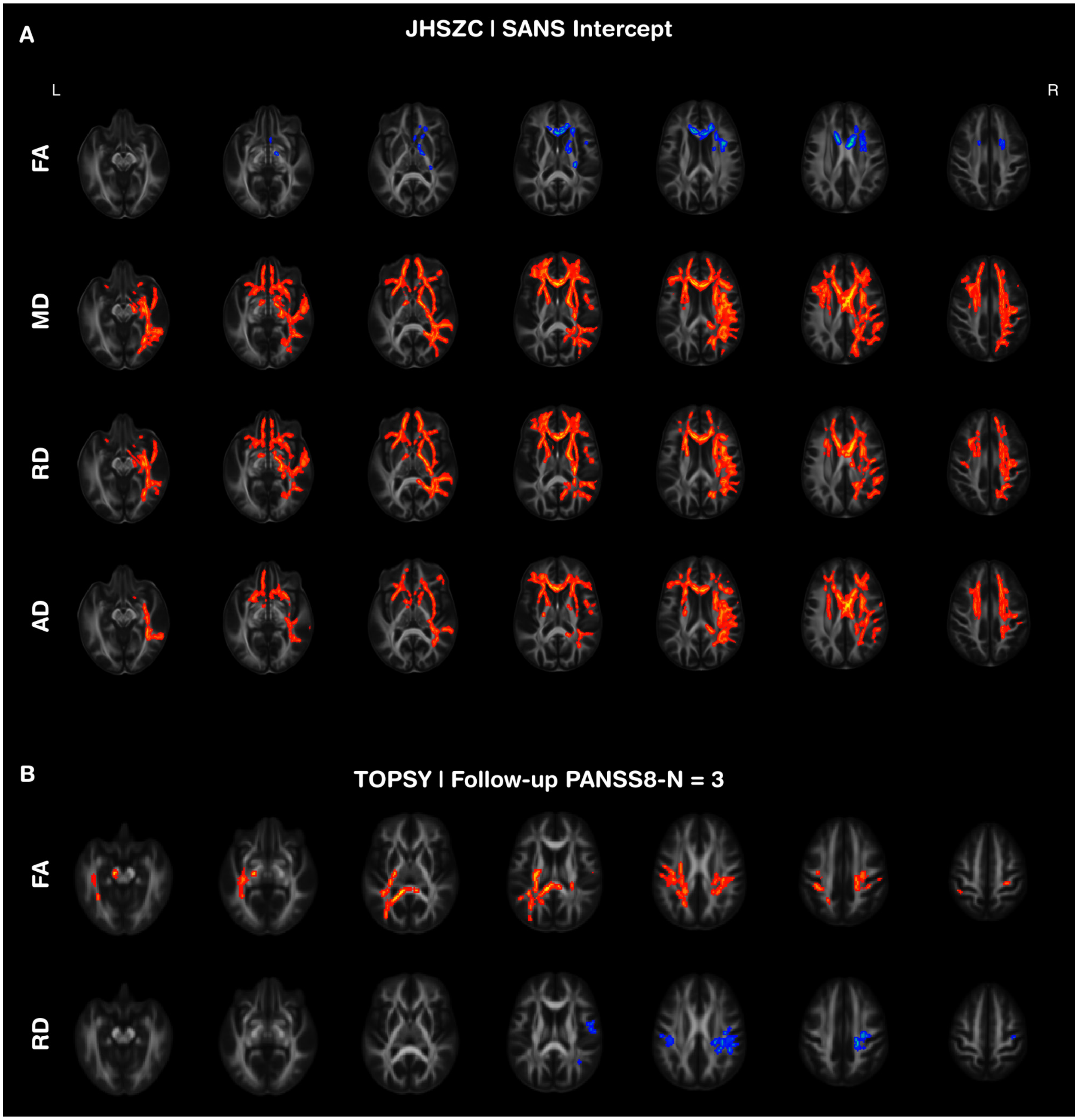

Figure S6: Regions associated with negative symptoms. Displayed clusters significantly correlate with the metric of interest as determined using TFCE (10,000 samples, FWER < 0.05*). Clusters are localized to the TBSS-derived FA skeleton and inflated for visualization. A: Measures in JHSCZ patients compared with the SANS intercept (first order linear model with baseline session as 0). B: Effect of PANSS8-N remission (follow-up PANSS8-N score of 3) on microstructure in TOPSY patients.*

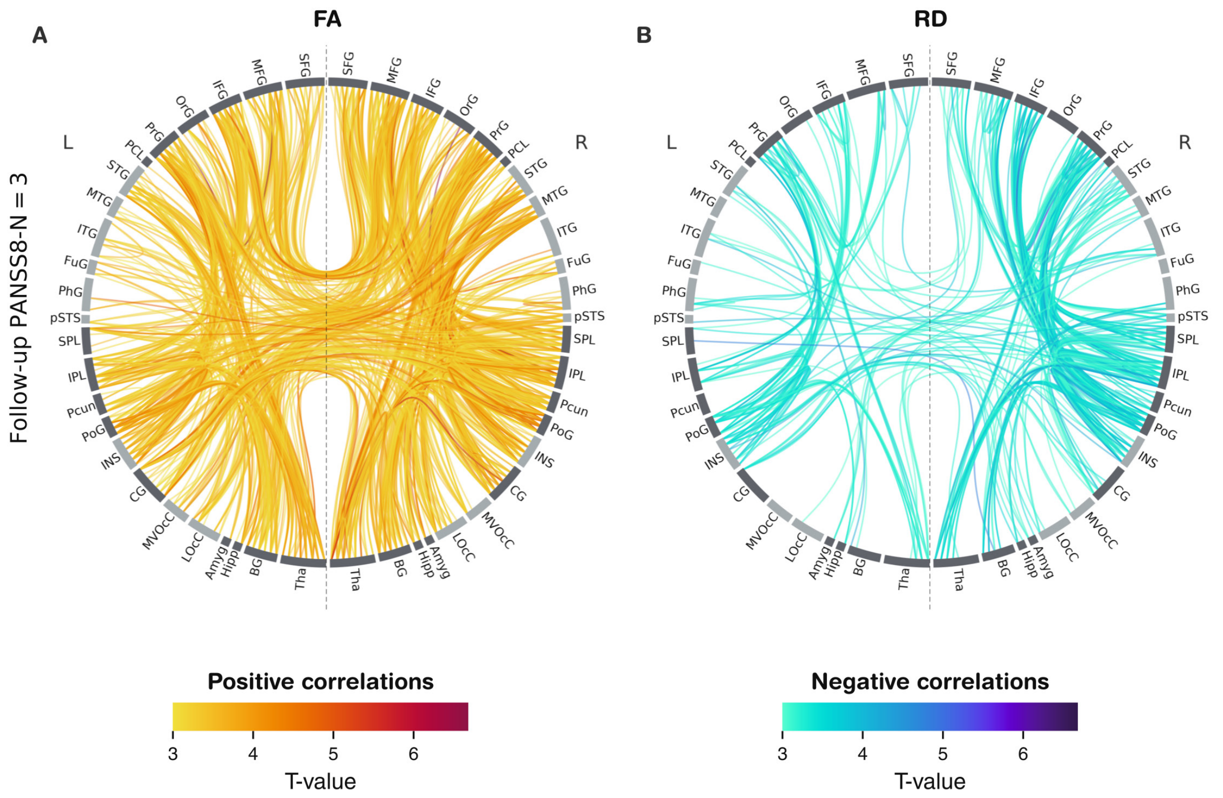

Figure S7: Correlations between DTI parameters and the PANSS8-N follow-up score in the TOPSY dataset. DTI measures were averaged across sessions per subject. Lines represent connections significantly correlated with PANSS8-N remission, as determined using NBS (10,000 samples, $T_{thresh} = 3$*,* $FWER < 0.05$*). Gyral abbreviations are given in Table S1. Subnetwork size and p-values are given in Table S11.* ***A*** *FA;* ***B*** *RD.*

2 CORTICAL ATLASES

Table S1: Abbreviations of cortical regions.

Gyrus Abbr	Gyrus	Lobe
SFG	Superior frontal gyrus	Frontal lobe
MFG	Middle frontal gyrus	Frontal lobe
IFG	Inferior frontal gyrus	Frontal lobe
OrG	Orbital gyrus	Frontal lobe
PrG	Precentral gyrus	Frontal lobe
PCL	Paracentral lobule	Frontal lobe
STG	Superior temporal gyrus	Temporal lobe
MTG	Middle temporal gyrus	Temporal lobe
ITG	Inferior temporal gyrus	Temporal lobe
FuG	Fusiform gyrus	Temporal lobe
PhG	Parahippocampal gyrus	Temporal lobe
pSTS	Posterior superior temporal sulcus	Temporal lobe
SPL	Superior parietal lobule	Parietal lobe
IPL	Inferior parietal lobule	Parietal lobe
Pcun	Precuneus	Parietal lobe
PoG	Postcentral gyrus	Parietal lobe
INS	Insular gyrus	Insular lobe
CG	Cingulate gyrus	Limbic lobe
MVOcC	MedioVentral occipital cortex	Occipital lobe
LOcC	Lateral occipital cortex	Occipital lobe
Amyg	Amygdala	Subcortical nuclei
Hipp	Hippocampus	Subcortical nuclei
BG	Basal ganglia	Subcortical nuclei
Tha	Thalamus	Subcortical nuclei

Table S2: ROIs used in study, with the four subgroupings of the JHU atlas.

Atlas	Group	Region
Core (JHU)	Association	External capsule
		Inferior fronto-occipital fasciculus
		Sagittal stratum
		Superior fronto-occipital fasciculus
		Superior longitudinal fasciculus
		Uncinate fasciculus
	Callosal	CC Body
		CC Genu
		CC Splenium
	Limbic	Cingulate gyrus
		Fornix
		Fornix (cres)
		Hippocampus
	Projection	Anterior corona radiata
		Cerebral peduncle
		Corticospinal tract
		IC Anterior Limb
		IC Posterior Limb
		IC Retrolenticular
		Medial lemniscus
		Posterior corona radiata
		Posterior thalamic radiation
		Superior corona radiata
Peripheral		Anterior Lobe
		Frontal Lobe
		Limbic Lobe
		Occipital Lobe
		Parietal Lobe
		Posterior Lobe
		Temporal Lobe

3 DEMOGRAPHIC TABLES

Table S3: JHSZC demographic statistics: HC vs patient.

	Baseline	1yr	2yr
Sex (M/F)	$\chi^2(1) = 3.59$, $P = .058$	$\chi^2(1) = 3.33$, $P = .068$	$\chi^2(1) = 3.41$, $P = .065$
Age	$T(64) = 2.05$, $\mathbf{P = .045}$	$T(60) = 2.44$, $\mathbf{P = .018}$	$T(27) = 2.83$, $\mathbf{P = .0086}$
Ethnicity (B/EA/O/W)	$\chi^2(3) = 2.36$, $P = .5$	$\chi^2(3) = 3.43$, $P = .33$	$\chi^2(3) = 1.63$, $P = .65$
Handedness (R/L)	$\chi^2(1) = 0.00947$, $P = .92$	$\chi^2(1) = 0.14$, $P = .71$	$\chi^2(1) = 0.207$, $P = .65$
Smoker (Yes/No)	$\chi^2(1) = 1.56$, $P = .21$	$\chi^2(1) = 8.92$, $\mathbf{P = .0028}$	$\chi^2(1) = 4.42$, $\mathbf{P = .036}$
Cannabis (Yes/No)	$\chi^2(1) = 0.629$, $P = .43$	$\chi^2(1) = 0.972$, $P = .32$	$\chi^2(1) = 0.153$, $P = .7$

B=Black/African; EA=East Asian; O=Other/Unknown; W=White/European

Table S4: TOPSY demographic statistics.

	Baseline	6 mo
Sex (M/F)	$\chi^2(1) = 0.625$, $P = .43$	$\chi^2(1) = 0.625$, $P = .43$
Age	$T(32) = -0.508$, $P = .61$	$T(32) = -0.323$, $P = .75$
Ethnicity	$\chi^2(3) = 7.81$, $P = .05$	$\chi^2(3) = 7.81$, $P = .05$
Handedness (R/L/A)	$\chi^2(1) = 0$, $P = 1$	$\chi^2(1) = 0$, $P = 1$
Education	$T(32) = 2.39$, $\mathbf{P = .023}$	$T(32) = 2.44$, $\mathbf{P = .02}$
SES	$T(31) = -1.49$, $P = .15$	$T(31) = -1.49$, $P = .15$
CAST	$T(29) = -2.53$, $\mathbf{P = .017}$	
AUDIT-C	$T(27) = -0.794$, $P = .43$	
Smoker (yes/no)	$\chi^2(1) = 2.73$, $P = .098$	$\chi^2(1) = 0.0145$, $P = .9$
Cannabis (yes/no)	$\chi^2(1) = 1.91$, $P = .17$	$\chi^2(1) = 1.91$, $P = .17$
SOFAS	$T(30) = 9.8$, $\mathbf{P < .001}$	$T(24) = 3.11$, $\mathbf{P = .0048}$

B=Black/African; C=Caribbean/North American Black; EA=East Asian; W=White/European; CAST=Cannabis Abuse Screening Test; SES=Socioeconomic status; AUDIT-C=Alcohol Use Disorders Identification Test

Table S5: JHSZC dropout demographics.

	Healthy Control			Early Psychosis		
	Dropout (n=54)	Included (n=42)	Dropout vs Included	Dropout (n=41)	Included (n=28)	Dropout vs Included
Sex (M/F)	19/35	22/20	$\chi^2(1)$ = 2.2, P = .14	32/9	23/5	$\chi^2(1)$ = 0.0122, P = .91
Age	23.24 (4.29)	23.93 (3.39)	$T(94)$ = -0.852, P = .4	22.12 (4.57)	21.86 (3.73)	$T(67)$ = 0.254, P = .8
Ethnicity	B=38 EA=0 H=3 O=1 W=12	B=23 EA=2 H=0 O=2 W=15	$\chi^2(4)$ = 7.98, P = .092	B=18 EA=2 H=0 O=4 W=17	B=21 EA=0 H=0 O=1 W=6	$\chi^2(3)$ = 7.09, P = .069
Handedness (R/L)	51/3	35/7	$\chi^2(1)$ = 2.05, P = .15	39/2	24/4	$\chi^2(1)$ = 0.859, P = .35
Smoker (Yes/No)	1/53	3/39	$\chi^2(1)$ = 0.596, P = .44	16/25	7/21	$\chi^2(1)$ = 0.909, P = .34
Cannabis (Yes/No)	4/50	3/39	$\chi^2(1)$ = 0, P = 1	13/28	4/24	$\chi^2(1)$ = 1.86, P = .17
Duration of Illness (weeks)				69.33 (69.33)	67.17 (53.08)	$T(67)$ = 0.196, P = .85
CPZ (mg)				262.55 (292.04)	250.36 (217.96)	$T(67)$ = 0.188, P = .85
SAPS				3.83 (3.40)	4.44 (4.06)	$T(65)$ = -0.676, P = .5
SANS				9.10 (5.70)	8.26 (4.14)	$T(65)$ = 0.658, P = .51

[1] *Median (IQR)*

B=Black/African; EA=East Asian; O=Other/Unknown; W=White/European; CPZ=chlorpromazine equivalent dose; SAPS=Scale for assessment of positive symptoms; SANS=Score for assessment of negative symptoms

Table S6: TOPSY dropout demographics.

	Healthy Control			First Episode Psychosis		
	Dropout (n=24)	Included (n=15)	Dropout vs Included	Dropout (n=52)	Included (n=19)	Dropout vs Included
Sex (M/F)	16/8	10/5	$\chi^2(1) = 0$, *P* = 1	41/10	16/3	$\chi^2(1) =$ 0.00039, *P* = .98
Age	21.83 (3.80)	21.73 (2.99)	*T*(37) = 0.0865, *P* = .93	22.74 (4.07)	22.53 (5.42)	*T*(67) = 0.177, *P* = .86
Ethnicity	EA=5<br>M=0<br>SA=3<br>W=12	EA=0<br>M=4<br>SA=0<br>W=10	$\chi^2(3) = 11.5$, ***P* = .0094**	B=5<br>C=1<br>EA=0<br>I=0<br>M=3<br>O=0<br>SA=4<br>W=24	B=2<br>C=1<br>EA=0<br>I=0<br>M=0<br>O=0<br>SA=0<br>W=16	$\chi^2(4) = 4.57$, *P* = .33
Handedness (R/L/A)	22/0/2	14/0/1	$\chi^2(1) = 0$, *P* = 1	41/1/10	18/0/1	$\chi^2(2) = 2.54$, *P* = .28
Education	14.04 (2.23)	14.27 (2.02)	*T*(36) = -0.313, *P* = .76	12.62 (2.06)	12.95 (1.18)	*T*(67) = -0.652, *P* = .52
SES	3.09 (1.20)	3.20 (1.57)	*T*(36) = -0.251, *P* = .8	3.49 (1.60)	3.89 (1.08)	*T*(57) = -0.97, *P* = .34
CAST	7.17 (3.28)	7.00 (3.87)	*T*(37) = 0.144, *P* = .89	12.44 (5.95)	12.12 (6.90)	*T*(59) = 0.177, *P* = .86
AUDIT-C	3.38 (2.24)	2.87 (2.26)	*T*(37) = 0.686, *P* = .5	1.60 (2.19)	3.79 (3.83)	*T*(54) = -2.65, ***P* = .01**
Smoker (yes/no)	0/24	1/14	$\chi^2(1) = 0.0577$, *P* = .81	12/40	7/12	$\chi^2(1) = 0.735$, *P* = .39
Cannabis (yes/no)	7/17	5/10	$\chi^2(1) = 0$, *P* = 1	33/15	12/7	$\chi^2(1) =$ 0.0227, *P* = .88
SOFAS	82.52 (3.14)	81.08 (6.24)	*T*(32) = 0.9, *P* = .37	39.71 (12.20)	42.00 (13.37)	*T*(69) = -0.682, *P* = .5

	Healthy Control			First Episode Psychosis		
	Dropout (n=24)	Included (n=15)	Dropout vs Included	Dropout (n=52)	Included (n=19)	Dropout vs Included
Duration of Illness (weeks)				139.00 (238.00)[1]	104.00 (132.50)[1]	*T*(53) = 1.02, *P* = .31
Antipsych. Day of Scan (DDD)				0.20 (0.66)[1]	0.00 (0.33)[1]	*T*(66) = 0.551, *P* = .58
Antipsych. Lifetime (DDD-days)				0.90 (3.50)[1]	0.00 (1.25)[1]	*T*(67) = 1.29, *P* = .2
PANSS-8 Total				25.71 (7.70)	24.68 (5.53)	*T*(62) = 0.526, *P* = .6
PANSS-8 Positive				12.18 (3.14)	12.53 (2.82)	*T*(62) = -0.417, *P* = .68
PANSS-8 Negative				8.00 (4.66)	6.26 (3.31)	*T*(63) = 1.47, *P* = .15
PANSS-8 General				5.57 (2.24)	5.89 (2.54)	*T*(63) = -0.519, *P* = .61

[1] *Median (IQR)*

B=Black/African; C=Caribbean/North American Black; EA=East Asian; W=White/European; DDD-days=Defined daily dose × Days; CDS=Calgary Depression Scale; CAST=Cannabis Abuse Screening Test; PANSS=Positive and Negative Symptom Scale; SES=Socioeconomic status; AUDIT-C=Alcohol Use Disorders Identification Test; SOFAS=Social and Occupational Functioning Assessment Scale

Table S7: JHSZC age-matched demographics (age < 24yr).

	Healthy Control (n=19)			Early Psychosis (n=21)		
	Baseline (n=19)	1yr (n=13)	2yr (n=4)	Baseline (n=17)	1yr (n=14)	2yr (n=6)
Sex (M/F)	9/10	5/8	1/3	14/3	11/3	5/1
Age	21.05 (1.58)	21.85 (1.34)	21.00 (0.82)	20.18 (2.35)	20.43 (2.41)	20.00 (2.68)
Ethnicity	B=11<br>EA=1<br>O=1<br>W=6	B=8<br>EA=0<br>O=1<br>W=4	B=2<br>EA=1<br>O=1<br>W=0	B=13<br>EA=0<br>O=0<br>W=4	B=13<br>EA=0<br>O=0<br>W=1	B=5<br>EA=0<br>O=0<br>W=1
Handedness (R/L)	17/2	11/2	4/0	15/2	12/2	6/0
Smoker (Yes/No)	0/19	0/13	0/4	4/13	**6/8**	2/4
Cannabis (Yes/No)	1/18	1/12	0/4	3/14	3/11	0/6
Duration of Illness (weeks)				56.33 (82.33)	106.17 (70.42)	160.33 (29.25)
CPZ (mg)				248.24 (220.29)	312.86 (294.31)	400.00 (238.75)
SAPS				4.06 (4.77)	4.75 (2.83)	3.83 (5.95)
SANS				7.62 (4.15)	6.17 (3.97)	6.33 (6.65)

[1] *Median (IQR)*

***Bold** values under Early Psychosis are significantly different relative to the same session in Healthy Controls.*

B=Black/African; EA=East Asian; O=Other/Unknown; W=White/European; CPZ=chlorpromazine equivalent dose; SAPS=Scale for assessment of positive symptoms; SANS=Score for assessment of negative symptoms

Table S8: JHSZC age-matched demographics statistics (age < 24yr).

	Baseline	1yr	2yr
Sex (M/F)	$\chi^2(1) = 5.05$, $\boldsymbol{P = .025}$	$\chi^2(1) = 2.98$, $P = .084$	$\chi^2(1) = 1.41$, $P = .24$
Age	$T(38) = 1.19$, $P = .24$	$T(25) = 1.87$, $P = .074$	$T(8) = 0.711$, $P = .5$
Ethnicity (B/EA/O/W)	$\chi^2(3) = 3.59$, $P = .31$	$\chi^2(2) = 3.96$, $P = .14$	$\chi^2(3) = 4.05$, $P = .26$
Handedness (R/L)	$\chi^2(1) = 0$, $P = 1$	$\chi^2(1) = 0$, $P = 1$	$\chi^2(0) = 0$, $P = 1$
Smoker (Yes/No)	$\chi^2(1) = 4.34$, $\boldsymbol{P = .037}$	$\chi^2(1) = 4.9$, $\boldsymbol{P = .027}$	$\chi^2(1) = 0.234$, $P = .63$
Cannabis (Yes/No)	$\chi^2(1) = 0.178$, $P = .67$	$\chi^2(1) = 0.213$, $P = .64$	$\chi^2(0) = 0$, $P = 1$

B=Black/African; EA=East Asian; O=Other/Unknown; W=White/European

4 ANALYSIS TABLES

Table S9: DTI measures in TOPSY patients with a follow-up PANSS8-N score of 3 versus higher than 3.

	$N_{int} \sim -FA$		$N_{int} \sim MD$		$N_{int} \sim RD$		$N_{int} \sim AD$	
Region	$T(17)$	P_{corr}	$T(17)$	P_{corr}	$T(17)$	P_{corr}	$T(17)$	P_{corr}
White Matter	2.4	**.013**	-1.	.16	-1.5	.071	0.43	.66
Core White Matter	2.	**.029**	-0.54	.3	-1.4	.097	1.8	.96
Peripheral White Matter	2.6	**.021**	-1.3	.22	-1.6	.097	-0.31	.76
Limbic Tracts	2.5	**.046**	-0.84	.41	-1.8	.21	1.9	.99
Frontal Lobe	2.7	**.046**	-1.2	.38	-1.7	.21	-0.24	.98
Posterior Lobe	2.6	**.046**	-1.	.38	-1.4	.21	-0.28	.98

Statistics computed using paired, 1-tailed T-tests after regressing age and sex. Bold results indicate significant results (only rows with at least one such result are shown). All p-values corrected using FDR with comparisons in the same hierarchical level.

Table S10: Microstructural measures versus SANS intercept in JHSZC patients.

Region	$N_{int} \sim -FA$		$N_{int} \sim MD$		$N_{int} \sim RD$		$N_{int} \sim AD$	
	$T(24)$	P_{corr}	$T(24)$	P_{corr}	$T(24)$	P_{corr}	$T(24)$	P_{corr}
White Matter	-1.6	.06	3.1	**.003**	2.8	**.006**	2.6	**.007**
Core White Matter	-1.6	.079	2.6	**.008**	2.3	**.016**	2.5	**.01**
Peripheral White Matter	-1.5	.079	3.2	**.004**	2.9	**.009**	2.6	**.01**
Association Tracts	-0.65	.44	2.8	**.014**	2.1	.051	3.1	**.016**
Cerebellar Tracts	-0.044	.53	1.1	.17	0.51	.33	1.6	.13
Projection Tracts	-1.8	.23	2.6	**.021**	2.4	**.04**	2.3	.051
Frontal Lobe	-2.2	.23	3.3	**.009**	3.	**.014**	3.1	**.016**
Posterior Lobe	-1.4	.23	2.9	**.014**	3.	**.014**	1.8	.11
Temporal Lobe	-1.2	.26	3.5	**.009**	3.	**.014**	2.4	.051
External capsule	-1.3	.31	3.3	**.018**	2.7	.052	3.1	**.047**
Inferior fronto-occipital fasciculus	-0.67	.32	2.8	**.033**	1.7	.15	2.2	.095
Superior longitudinal fasciculus	0.44	.76	2.	.078	0.61	.33	2.9	**.047**
CC Genu	-2.1	.27	2.6	**.041**	2.5	.053	2.	.095
Hippocampus	-1.5	.31	2.5	**.041**	2.5	.053	1.5	.16
Anterior corona radiata	-1.4	.31	3.1	**.021**	2.7	.052	2.5	.087
Corticospinal tract	-0.24	.49	2.3	**.049**	1.5	.16	1.9	.095
IC Anterior Limb	-3.	.082	3.5	**.018**	3.7	**.013**	2.	.095
Medial lemniscus	-1.1	.31	1.6	.12	1.6	.16	0.66	.36
Superior corona radiata	-1.	.31	2.4	**.041**	2.1	.099	1.9	.095

Intercept computed for each subject by fitting a first-order linear model. Statistics computed using unpaired, 1-tailed T-test after regressing age and sex. Bold results indicate significant results (only rows with at least one such result are shown). All p-values corrected using FDR with comparisons in the same hierarchical level.

Table S11: Subnetworks with a significant association between DTI parameter and a follow-up PANSS8-N score of 3.

Param	# Connections	P_{corr}
RD	486	.029
FA	1278	.007

5 Supplementary Methods

5.1 Inclusion Criteria

5.1.1 Johns Hopkins Schizophrenia Center (JHSZC)

Inclusion criteria were: 1) between 13 and 35 years old; 2) no history of traumatic brain injury, cancer, abnormal bleeding, viral infection, neurologic disorder, or intellectual disability; 3) no drug or alcohol abuse (not including cannabis or synthetic cannabinoid receptor agonists) in the past three years; 4) no illicit drug use in the past two months. Patients were within 24 months of the onset of psychotic symptoms as assessed by study team psychiatrists using the Structured Clinical Interview for DSM-IV (SCID) and collateral information from available medical records.

5.1.2 Tracking Outcomes in Psychosis (TOPSY)

Inclusion criteria for first-episode psychosis (FEP) patients were: individuals experiencing their first psychotic episode, with no more than 14 days of cumulative lifetime antipsychotic exposure, no major head injuries, no known neurological disorders, and no concurrent substance use disorder. Participants were not explicitly instructed to abstain from substances, and patients on non-antipsychotic prescription medication were not excluded. Patient diagnosis was established using a best estimate procedure (1) based on DSM-5 criteria and confirmed after 6 months of treatment. Two patients with major depressive disorder and two with bipolar disorder were excluded from analysis.

Healthy controls (HCs) were recruited through posters and word-of-mouth advertising. They had no personal history of mental illness, no current use of medications, and no first-degree relatives with a history of psychotic disorders. HCs were group matched to the FEP cohort for age and sex. Like their FEP counterparts, those with a history of substance use disorders in the past twelve months, significant head injury, or neurological disorders were excluded.

5.2 Template Generation

Fractional anisotropy (FA) maps were non-linearly registered to a common template corresponding to the average space of all FA images. This template was computed with an in-house implementation of the iterative algorithm described by Avants *et al.* (2), using *greedy* (3) to compute each transformation.

5.3 Parcellations

Global surface averages were computed by averaging across all vertices for each parameter map, per hemisphere. The Desikan-Killiany atlas computed by *fastsurfer* was used to parcellate the surface data (Table S1). Hemispheres were kept separate throughout the analysis.

A composite, hierarchical atlas was constructed using the Johns Hopkins University (JHU) atlas distributed with *FSL* (4), which capture core white matter regions, and the Talairach lobe segmentation (5), which capture peripheral regions. Both atlases were first intersected with the skeletonized image, and the lobe segmentation was used to define a peripheral atlas by subtracting the JHU atlas. Mathematically:

$$\begin{aligned} A^{JHU} &= JHU \cap M^{skeleton} \\ A^{Lobe} &= Lobe \ \cap M^{skeleton} \\ A_{ijk}^{Periph} &= \begin{cases} A_{ijk}^{Lobe} & A_{ijk}^{JHU} = 0 \\ 0 & A_{ijk}^{JHU} \neq 0 \end{cases} \end{aligned}$$

where A_{ijk} is the ijkth voxel of the indicated atlas. Homologous ROIs across the two hemispheres were merged.

To increase our sensitivity to local and global changes, the ROIs were organized into a hierarchical system (6). Multiple comparisons were corrected separately across each level using the Benjamini-Hochberg false discovery rate procedure (7). Four hierarchical levels were defined (Table S2):

1. The global mean (1 ROI)
2. Mean of all voxels in the peripheral atlas and of all voxels in the JHU atlas (2 ROIs)
3. Peripheral atlas and JHU atlas merged into 4 tract groupings (11 ROIs)
4. JHU atlas (23 ROIs)

5.4 Tract-Based Spatial Statistics (TBSS)

TBSS was used to further characterize the relationship between clinical score intercepts and slopes and diffusion tensor imaging (DTI) parameters. As with the ROI analysis, the DTI parameter maps were averaged by subject across sessions and were projected to an FA-derived skeleton created with the TBSS pipeline from FSL (8). The model was analyzed with *FSL randomise* (9) using threshold-free cluster enhancement-correction and 10,000 permutations, thresholding at a corrected p-value of 0.05. Sex and age were regressed as nuisance variables. One-way comparisons were performed as in the ROIs.

5.5 Network based statistic (NBS)

NBS was used to additionally analyze the relation between clinical score intercept, slope, and remission in the TOPSY dataset. This analysis was not performed for JHSZC because of the low angular resolution of the diffusion data.

5.5.1 Anatomical Preprocessing

Segmentation of anatomical images and construction of the cortical surface mesh was performed using *FastSurfer* (10–12). These outputs were further processed with *ciftify* (13), an implementation of the Human Connnectome Project minimal preprocessing workflow (14).

Images were registered to the *MNI152NLin6Asym* (15) template space, and meshes were registered to the *fsLR-32k* template space (16).

Tractography was performed using the *MRtrix3* (17) software suite. Constrained spherical deconvolution was performed using the dhollander algorithm to estimate the response functions for white matter, grey matter and cerebrospinal fluid (CSF) (18,19). Single shell 3 tissue constrained spherical deconvolution (SS3T-constrained spherical deconvolution), as implemented in *MRtrix3Tissue* (https://3tissue.github.io/), was performed to obtain white matter-like fibre orientation distributions (FODs) as well as grey matter-like and CSF-like compartments in all voxels (20). Residual intensity inhomogeneities were corrected with *mtnormalise* (21,22). Tractography was performed using the iFOD2 algorithm (23) and anatomically constrained tractography (ACT) (24), with 10,000,000 streamlines (25), an FOD amplitude cut-off of 0.06, a minimum streamline length of 4mm, and a maximum streamline length of 250mm. The anatomical segmentation used for ACT was obtained using *SynthSeg* (26).

Subject anatomical scans were parcellated using the Brainnetome atlas (27), which were used to derive weighted connectivity matrices based on the tractography data. Average FA, mean diffusivity, radial diffusivity, axial diffusivity, and the log-transformed spherical-deconvolution informed filtering of tractograms-weighted streamline count were used as weights. Sampled connectomes were then averaged together by subject across sessions for analysis.

5.5.2 Statistics

NBS was performed with extent-based cluster sizes, a T threshold of 3.0, 10,000 iterations, and corrected p-value threshold of 0.05. Sex and age were regressed as nuisance variables (28). One-way comparisons were performed as in the ROIs.

6 References

1. Leckman JF, Sholomskas D, Thompson WD, Belanger A, Weissman MM (1982): Best estimate of lifetime psychiatric diagnosis: a methodological study. *Archives of General Psychiatry* 39: 879–883.

2. Avants BB, Yushkevich P, Pluta J, Minkoff D, Korczykowski M, Detre J, Gee JC (2010): The Optimal Template Effect in Hippocampus Studies of Diseased Populations. *NeuroImage* 49: 2457.

3. Yushkevich PA, Pluta J, Wang H, Wisse LEM, Das S, Wolk D (2016): IC-P-174: Fast Automatic Segmentation of Hippocampal Subfields and Medial Temporal Lobe Subregions In 3 Tesla and 7 Tesla T2-Weighted MRI. *Alzheimer's & Dementia* 12: P126–P127.

4. Mori S, Wakana S, Zijl PCM van, Nagae-Poetscher LM (2005): *MRI Atlas of Human White Matter*. Elsevier.

5. Talairach J, Szikla G (1980): Application of stereotactic concepts to the surgery of epilepsy. *Acta Neurochirurgica Supplementum* 30: 35–54.

6. Simmonds DJ, Hallquist MN, Asato M, Luna B (2014): Developmental stages and sex differences of white matter and behavioral development through adolescence: A longitudinal diffusion tensor imaging (DTI) study. *NeuroImage* 92: 356–368.

7. Benjamini Y, Hochberg Y (1995): Controlling the false discovery rate: A practical and powerful approach to multiple testing. *Journal of the Royal statistical society: series B (Methodological)* 57: 289–300.

8. Smith SM, Jenkinson M, Johansen-Berg H, Rueckert D, Nichols TE, Mackay CE, *et al.* (2006): Tract-based spatial statistics: voxelwise analysis of multi-subject diffusion data. *NeuroImage* 31: 1487–1505.

9. Winkler AM, Ridgway GR, Webster MA, Smith SM, Nichols TE (2014): Permutation Inference for the General Linear Model. *NeuroImage* 92: 381–397.

10. Faber J, Kügler D, Bahrami E, Heinz L-S, Timmann D, Ernst TM, *et al.* (2022): CerebNet: A Fast and Reliable Deep-Learning Pipeline for Detailed Cerebellum Sub-Segmentation. *NeuroImage* 264: 119703.

11. Henschel L, Conjeti S, Estrada S, Diers K, Fischl B, Reuter M (2020): FastSurfer - A Fast and Accurate Deep Learning Based Neuroimaging Pipeline. *NeuroImage* 219: 117012.

12. Henschel L, Kügler D, Reuter M (2022): FastSurferVINN: Building Resolution-Independence into Deep Learning Segmentation methods—A Solution for HighRes Brain MRI. *NeuroImage* 251: 118933.

13. Dickie EW, Smith DE, Mathu-M, lgrennan, Jeyachandra J, delaneyjohnston, Gorgolewski C (2019, August 16): Edickie/ciftify: Fix to ciftify_meants and new ciftify_dlabel_to_vol script. Zenodo. https://doi.org/10.5281/zenodo.3369937

14. Glasser MF, Sotiropoulos SN, Wilson JA, Coalson TS, Fischl B, Andersson JL, *et al.* (2013): The minimal preprocessing pipelines for the Human Connectome Project. *NeuroImage* 80: 105–124.

15. Evans AC, Janke AL, Collins DL, Baillet S (2012): Brain Templates and Atlases. *NeuroImage* 62: 911–922.

16. Van Essen DC, Glasser MF, Dierker DL, Harwell J, Coalson T (2012): Parcellations and Hemispheric Asymmetries of Human Cerebral Cortex Analyzed on Surface-Based Atlases. *Cerebral Cortex* 22: 2241–2262.

17. Tournier J-D, Smith R, Raffelt D, Tabbara R, Dhollander T, Pietsch M, *et al.* (2019): MRtrix3: A Fast, Flexible and Open Software Framework for Medical Image Processing and Visualisation. *NeuroImage* 202: 116137.

18. Dhollander T, Raffelt D, Connelly A (2016): Unsupervised 3-tissue response function estimation from single-shell or multi-shell diffusion MR data without a co-registered T1 image.

19. Dhollander T, Mito R, Raffelt D, Connelly A (2019): Improved white matter response function estimation for 3-tissue constrained spherical deconvolution.

20. Dhollander T, Connelly A (2016): A novel iterative approach to reap the benefits of multi-tissue CSD from just single-shell (+b=0) diffusion MRI data.

21. Raffelt D, Dhollander T, Tournier J-D, Tabbara R, Smith R, Pierre E, Connelly A (2017): Bias Field Correction and Intensity Normalisation for Quantitative Analysis of Apparent Fibre Density.

22. Dhollander T, Tabbara R, Rosnarho-Tornstrand J, Tournier J-D, Raffelt D, Connelly A (2021): Multi-tissue log-domain intensity and inhomogeneity normalisation for quantitative apparent fibre density.

23. Tournier J-D, Calamante F, Connelly A (2010): Improved probabilistic streamlines tractography by 2nd order integration over fibre orientation distributions. *Proc Intl Soc Mag Reson Med (ISMRM)* 18.

24. Smith RE, Tournier J-D, Calamante F, Connelly A (2012): Anatomically-Constrained Tractography: Improved Diffusion MRI Streamlines Tractography Through Effective Use of Anatomical Information. *NeuroImage* 62: 1924–1938.

25. Newlin NR, Rheault F, Schilling KG, Landman BA (2023): Characterizing Streamline Count Invariant Graph Measures of Structural Connectomes. *Journal of Magnetic Resonance Imaging* 58: 1211–1220.

26. Billot B, Greve DN, Puonti O, Thielscher A, Van Leemput K, Fischl B, *et al.* (2023): SynthSeg: Segmentation of Brain MRI Scans of Any Contrast and Resolution Without Retraining. *Medical Image Analysis* 86: 102789.

27. Fan L, Li H, Zhuo J, Zhang Y, Wang J, Chen L, *et al.* (2016): The Human Brainnetome Atlas: A New Brain Atlas Based on Connectional Architecture. *Cerebral Cortex* 26: 3508–3526.

28. Zalesky A, Fornito A, Bullmore ET (2010): Network-based statistic: identifying differences in brain networks. *NeuroImage* 53: 1197–1207.
